# Aberrant Mucin5B expression in lung adenocarcinomas detected by iTRAQ labeling quantitative proteomics and immunohistochemistry

**DOI:** 10.1186/1559-0275-10-15

**Published:** 2013-11-01

**Authors:** Yan Li, Xiangchun Wang, MingHui Ao, Edward Gabrielson, Frederic Askin, Hui Zhang, Qing Kay Li

**Affiliations:** 1 Department of Pathology, The Johns Hopkins Medical Institutions, Baltimore, MD 21287, USA; 2 Department of Pathology, The Johns Hopkins Bayview Medical Center, 4940 Eastern Ave., AA Building, Room 154, Baltimore, MD 21224, USA; 3 Current address: Institute of Biophysics, Room 9214, 15 Datun Road, Room 9214, Chanyang, Beijing 100101, P. R. China

**Keywords:** Non-small cell lung cancer, Lung adenocarcinoma, Expression of mucin5B, Quantitative proteomics and iTRAQ labeling, Immunohistochemistry

## Abstract

**Background:**

Lung cancer is the number one cause of cancer-related deaths in the United States and worldwide. The complex protein changes and/or signature of protein expression in lung cancer, particularly in non-small cell lung cancer (NSCLC) has not been well defined. Although several studies have investigated the protein profile in lung cancers, the knowledge is far from complete. Among early studies, mucin5B (MUC5B) has been suggested to play an important role in the tumor progression. MUC5B is the major gel-forming mucin in the airway. In this study, we investigated the overall protein profile and MUC5B expression in lung adenocarcinomas, the most common type of NSCLCs.

**Methods:**

Lung adenocarcinoma tissue in formalin-fixed paraffin-embedded (FFPE) blocks was collected and microdissected. Peptides from 8 tumors and 8 tumor-matched normal lung tissue were extracted and labeled with 8-channel iTRAQ reagents. The labeled peptides were identified and quantified by LC-MS/MS using an LTQ Orbitrap Velos mass spectrometer. MUC5B expression identified by iTRAQ labeling was further validated using immunohistochemistry (IHC) on tumor tissue microarray (TMA).

**Results:**

A total of 1288 peptides from 210 proteins were identified and quantified in tumor tissues. Twenty-two proteins showed a greater than 1.5-fold differences between tumor and tumor-matched normal lung tissues. Fifteen proteins, including MUC5B, showed significant changes in tumor tissues. The aberrant expression of MUC5B was further identified in 71.1% of lung adenocarcinomas in the TMA.

**Discussions:**

A subset of tumor-associated proteins was differentially expressed in lung adenocarcinomas. The differential expression of MUC5B in lung adenocarcinomas suggests its role as a potential biomarker in the detection of adenocarcinomas.

## Introduction

Lung cancer is the number one cause of cancer-related deaths in the United States and worldwide [1]. Non-small cell lung cancer (NSCLC) accounts for approximately 80-85% of all lung cancers [1,2]. Among NSCLC subtypes, adenocarcinoma (approximately consisting of 60 to 70% of all NSCLC), has been markedly increasing in incidence over recent years [1,2]. Currently, targeted therapies have progressed rapidly, based upon the discovery of novel molecular markers such as mutations in *EGFR* (epidermal growth factor receptor), *KRAS* (V-Ki-ras2 Kirsten rat sarcoma viral oncogene homolog) genes and *ALK* (anaplastic lymphoma kinase) rearrangements [3-5], however, the overall progression-free survival rate of lung cancer patients is still suboptimal [6].

Lung cancer development is a multistep process characterized by genetic alterations and subsequently abnormal expression of cellular proteins [7,8]. During the process, cellular proteins, such as those associated with intracellular organelles or located on cell surfaces and secreted into the extracellular environment, play important biological roles in the regulation of cell growth and differentiation. In lung tissue, protein expression directly reflects the physiological and/or pathological status of the lung parenchyma [9-12]. Several recent studies have discovered that many proteins are differentially expressed in lung cancers [13-18]. The complex protein changes and/or signature of protein expression, particularly those associated with NSCLC, still need to be further defined.

Among these potential protein biomarkers, the abnormal expression of mucin proteins in lung cancers is particularly interesting. Mucin proteins are a family of high molecular weight glycoproteins, which are expressed by epithelial cells and/or goblet cells in the airway [19]. Both secretory and membrane-associated mucin proteins play important roles in the regulation of normal lung functions and are involved in many lung diseases [19,20]. For example, they are involved in the innate immune defense system of the lung to protect the airway against environmental toxins [19]. The overexpression of mucin proteins has been found in many chronic lung diseases, such as asthma, chronic obstructive pulmonary diseases and cystic fibrosis [19]. Although more than 20 mucin genes and related proteins are identified in human [19-21], the main mucin genes in lung trachoebronchial tree are *MUC5B*, *MUC5AC*, and *MUC4*[21]. An early study of airway mucin gene expression has demonstrated that *MUC5* was overexpressed in NSCLCs; and the elevated level of MUC5 was associated with the early recurrence of the tumor and poor prognosis of patients [22]. A recent study has shown that alteration in the expression of MUC1, MUC5AC, and MUC6 are correlated with p53 gene abnormalities in a subtype of lung adenocarcinomas [23]. However, potential role of MUC5B is still not fully understood in lung cancers.

Recent advances in proteomics have provided a novel approach to characterize the protein profile in lung cancer, such as high-content quantitative proteomics using LC-MS/MS (liquid chromatography tandem mass spectrometry) and iTRAQ (isobaric tags for relative and absolute quantitation) labeling [24,25]. These techniques have an increased sensitivity and throughput capability in accurate analysis of biological samples. For example, iTRAQ labeling technique, using stable isotope to label samples, allows for an accurate measurement of the peptide abundance in biological samples by direct comparison of light and heavy peptides in the same spectrum, whereas, the LC-MS/MS method determines the peptide abundance by spectral count based on the number of redundant spectra for each protein. The combination of these techniques could increase the detection of low abundance proteins in biological samples.

In this study, we investigated the protein profiles, including the expression of MUC5B in lung adenocarcinomas using highly sensitive iTRAQ labeling approach, and further validate our observations by immunohistochemistry (IHC) using lung adenocarcinoma tissue microarray (TMA). The purpose of our study is to investigate the expression of tumor-associated proteins and their potential roles in lung adenocarcinomas.

## Material and methods

### Materials

Sodium periodate (Bio-Rad, Hercules, CA), Tris (2-carboxyethyl) phosphine (TCEP) (Pierce, Rockford IL), sequencing grade trypsin (Promega, Madison, WI), C18 columns (Waters, Sep-Pak Vac), α-cyano-4-hydroxycinnasmic acid (CHCA) (Agilent, Palo Alto, CA), MALDI 4700 mass calibration standards (Applied Biosystems, Foster City, CA), and other chemicals were purchased from Sigma-Aldrich. iTRAQ reagent and mass calibration standards were from Applied Biosystems (Foster City, CA); BCA assay kit was from Pierce (Rockford, IL); SCX columns and C18 resin were from Sepax (Newark, DE).

### Lung cancer sample collection and tumor tissue microarray (TMA) construction

Eight lung adenocarcinomas, including four cases each of pathological stage 1 (pT1) and stage2 (pT2) tumors, and 8 cases of tumor-matched normal lung tissues were obtained from surgical resected specimens at the Johns Hopkins Hospital. All tumors and tumor-matched normal lung tissues were fixed in formalin and embedded in paraffin prior to proteomic analysis.

Tumor tissue microarrays (TMA) were constructed with independently collected 45 cases of lung adenocarcinomas. Represented on arrays were 24 cases of pT1, 18 cases of pT2, two cases of pT3 and one case of pT4 tumors. The TMA was prepared using core samples (1 mm diameter, 3 cores per case) of paraffin tissue blocks. The pathological stage and subtype of lung adenocarcinomas were classified according to AJCC (American Joint Committee on Cancer) staging manual [26], WHO (World Health Organization) and International Association for the Study of Lung Cancer/American Thoracic Society classification [2,27]. All tumor samples were annotated with available clinical information in a manner that protected the patient identity.

### Protein and peptide extraction from tumor tissue for proteomic analysis

For proteomic analysis, the formalin-fixed paraffin-embedded (FFPE) tumor tissue and tumor-matched normal lung tissue were cut into 10 micron section and placed on a glass slide without staining prior to microdissection and protein extraction.

To recover protein, hydration step was performed. The tumor tissue section on glass slide was incubated with xylene for 15 min twice, 100% ethanol for 5 min, 90% ethanol for 5 min, 80% ethanol for 5 min, then rinsed with distill H_2_O for 30 second twice. The rehydrated tumor tissue was microdissected and collected into a centrifuge vial with 25 μl of PBS buffer. Tumor tissue was sonicated for 5 min on an ice bath and centrifuged at 13,200 rpm for 5 min. The protein concentration in the supernatant was measured using BCA protein assay kit (Thermo Fisher Scientific Inc., Rockford, Illinois). For protein digestion, 10 μg of proteins (20 μl in volume) was first denatured in 90 μl of 8 M urea, 0.4 M NH4HCO3, and 0.1% SDS for 1 hour at 60°C. The proteins were then reduced by incubating with 10 μl of 120 mM Tris(2-carboxyethyl) phosphine for 30 min and alkylated by mixing with 10 μl of 160 mM iodoacetamide at room temperature for 30 min in the dark. Sample was diluted by 200 μl of trypsin digestion buffer (100 mM Tris–HCl, pH 7.5) containing 2 μg of trypsin at 37°C overnight. The digested peptides were purified with C18 desalting columns and dried using SpeedVac.

### iTRAQ labeling of peptides in lung adenocarcinoma

The iTRAQ (isobaric tags for relative and absolute quantitation) 8-plex reagent was dissolved in 70 μl of methanol. 10 μg of tryptic peptide of each sample was added into 20 μl of iTRAQ dissolution buffer, then mixed with 70 μl of iTRAQ 8-plex reagent and incubated for 1 hour at room temperature. After iTRAQ labeling, the reaction solution was cleaned up by SCX column. Then, labeled peptides were dried and resuspended into 10 μl of 0.4% acetic acid solution prior to mass spectrometry analysis.

### Mass spectrometry analysis

For protein quantification by spectral count, each peptide mixture was analyzed using LC-MS/MS (liquid chromatography tandem mass spectrometry) by the LTQ ion trap mass spectrometer (Thermo Finnigan, San Jose, CA). During the assay, 10 μl (10 μg) peptides were injected into a peptide cartridge packed with C18 resin, and then passed through a 10 cm × 75 μm i.d. microcapillary HPLC (μLC) column packed with C18 resin. The effluent from the μLC column entered an electrospray ionization source in which peptides were ionized and passed directly into the mass spectrometers. A linear gradient of acetonitrile from 5%–32% over 100 min at flow rate of approximately 300 nL/min was applied. During the LC-MS mode, data was acquired in the *m/z* range of 400 and 2000.

Mass spectrometry analysis was performed using a data dependent analysis of the top ten precursors and a dynamic exclusion of 30 seconds. The data was acquired at 30,000 resolution for the precursor scans and 7500 for MS/MS. Target values of 1e6 for MS and 1e5 for MS/MS were set with maximum injection time of 100 and 300 milliseconds, respectively. Data was acquired with Monoisotopic Precursor Selection (MIPS) and Predictive AGC enabled.

### Analysis of iTRAQ labeled peptides using HPLC-Orbitrap-MS platform

iTRAQ labeled peptide was analysed in the same setting as described above. Peptides identification by LC-MS was performed using an Orbitrap MS/MS (Thermo Fisher Scientific Inc., Rockford, Illinois) interfaced with a 2D nanoLCsystem (Eksigent, Dublin, California). 10 μl of pooled peptides (10 μg) were loaded on a self packed C18 column (75 μm ID × 10 cm, Magic C18 5 μm, 100A), and gradient eluted over 100 minutes at 300 nL/minute into the mass spectrometer. The HPLC mobile phase A and B were 0.2% formic acid in HPLC grade water and 0.2% formic acid in HPLC grade acetonitrile, respectively. The mobile phase B was increased from 5% to 40% in 90 min.

### Peptide identifications

The acquired data was searched against the Homo sapiens taxonomy of the RefSeq database (Version 40, 04/16/2010) using the Mascot (Matrix Science version 2.2.0) search algorithm within Proteome Discoverer (Thermo Scientific version 1.1). The data was searched with two missed cleavages allowed and a tolerance of 15 ppm on the precursors and 0.02 daltons on the fragment ions. Modifications allowed included carbamidomethyl of cysteines set to static. Data was also searched against a decoy database and filtered to a 1% false discovery rate (FDR).

### Immunohistochemistry (IHC)

IHC studies were performed using two lung adenocarcinoma TMAs. The sections were cut at 4 microns and deparaffinized prior to performing IHC. After treatment with antigen retrieval buffer (Dako, California) at 70°C for 40 minutes, slides were incubated with mouse monoclonal antibody anti-human MUC5B (clone 19.4E) at 1:200 dilutions for 1 hr. (Abcam, Cambridge, Massachusetts). After washing, a secondary antibody conjugated with peroxidase was applied to detect and visualize the specific antigen-antibody complexes using LASB System-HRP assay kit (Dako, California). Staining of MUC5B was scored semi-quantitatively using a four tier system: 0, undetectable (0% positive cells); 1+, focally positive (<10% positive cells); 2+, moderately positive (<50% positive cells), and 3+, intensely positive (more than 50% positive cells). Care was taken not to interpret entrapped normal bronchial epithelium or pulmonary macrophages as positive for tumor staining. Appropriate controls were also included in the assay.

Chi-square and Fisher’s test were used to calculate the *p* value. If the alpha value was less than 0.05, it was considered statistically significant (*p* < 0.05).

## Results

### Clinical information

**Figure 1:**
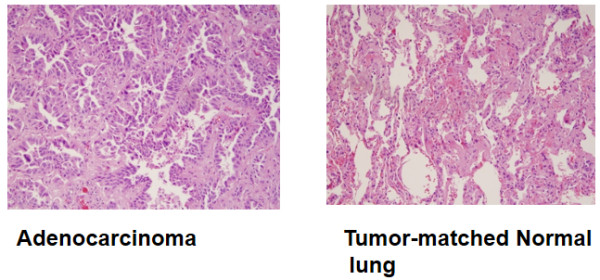
Histomorphology of lung adenocarcinomas.

A total of eight adenocarcinoma and tumor-matched normal control lung tissues were used for the proteomic analysis. The clinical information of these cases was summarized in Table 1. On lung adenocarcinoma TMA, a total of 45 cases were included. The subtypes of tumors were as follows: adenocarcinoma with papillary features (16 cases), adenocarcinoma NOS (13 cases), mucinous adenocarcinoma (6 cases), true papillary adenocarcinoma (6 cases) [28] and non-mucinous adenocarcinoma with lepidic pattern (formerly bronchioloalveolar adenocarcinoma, 4 cases). The representative morphology of a lung adenocarcinoma is shown in the Figure 1. In our study, the patients’ age ranged from 48 to 83 years with a median of 66.5 years. The male to female ratio was 1:1.37 (19 males and 26 females). 71.1% patients (32 of 45 cases) were current or ex-smokers. The average size of tumors was 3.56 cm (range from 0.7 to 12 cm).

**Table 1:** Clinical characterization of lung adenocarcinomas

**Cases**	**Age (years)**	**Sex**	**T size (cm)**	**T stage**	**Location of tumor**	**Surgery**
1	67	M	2.4	pT1	RLL	Lobectomy
2	69	F	1.5	pT1	RUL	Wedge resection
3	82	M	1.8	pT1	LUL	Lobectomy
4	55	F	1.5	pT1	RLL	Lobectomy
5	67	M	12.0	pT2	LLL	Lobectomy
6	64	F	9.0	pT2	RLL	Lobectomy
7	78	F	4.0	pT2	RUL	Lobectomy
8	77	M	2.5	pT2	LLL	Lobectomy

*T*: Tumor. *pT*: Pathological stage of tumor. *R*: Right. *L*: Left or lower or lobe. *U*: Upper.

### Protein identification and profile in lung adenocarcinoma tissues

**Figure 2:**
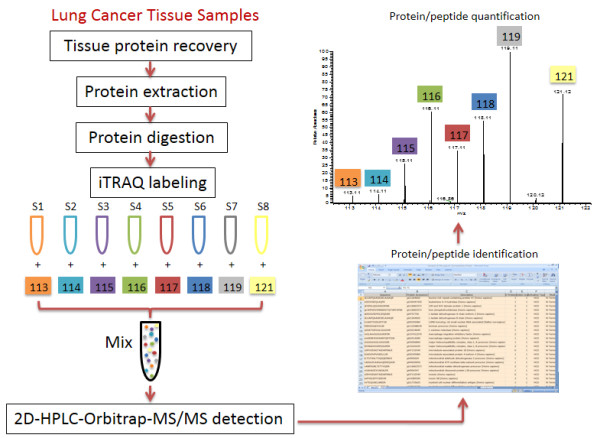
Diagram of work flow for determination of tumor-associated proteins in lung adenocarcinoma tissues.

The quantitative proteomic analyses are schematically illustrated in Figure 2 and consist of following steps: (1) tumor tissues were microdissected and proteins were extracted; (2) total proteins were further digested into peptides; (3) the peptides were labeled with 8-plex iTRAQ reagents; and (4) peptides were identified and quantified by LC-MS/MS, and spectral count. Peptides from four cases of pT1 tumor cases were labeled with iTRAQ channel 117, 118, 119, 120, while peptides from pT2 tumor cases were labeled with channel 113, 114, 115, and 116. The quantitative results were generated from the peak area of MS2 peaks of fragments (m/z from 113 to 121) of iTRAQ reagents. The LC-MS/MS method determines the peptide abundance by comparing the intensity of the same peptide peak in multiple LC-MS/MS runs. Quantitation of protein abundance by spectral count is based on the number of redundant spectra acquired for each protein from different samples in the LC-MS/MS analyses. We were able to identify and quantify 210 proteins using iTRAQ labeling [29].

We then determined and quantified the protein changes in different stage of tumors by iTRAQ. To determine the proteins with abundance changes in two stages, a histogram was used to generate the number of proteins in different abundance ratio. The threshold was set as < 0.7 and >1.3 for iTRAQ labeling. The means of pT1 and pT2 samples were used and the ratio of the means of pT1 to pT2 was calculated. Most of proteins (188 proteins, 89.5%) were distributed within one standard deviation from the mean and were considered as unchanged. Proteins that fell out of one standard deviation of the distribution curve were considered as with significant changes. A total of 22 proteins were identified between pT1 and pT2 tumors. These proteins were summarized in the Table 2.

**Table 2:** Proteins with differential expression in lung adenocarcinomas

**Accession**	**Proteins**	**MW [kDa]**	**Coverage**	**Ratio of T2/T1 tumor**
gi52426774	MHC, class II, DR alpha	28.6	5.51	3.109794973
gi110611233	Alpha 1 type XVIII collagen isoform 2	135.4	1.27	2.876066498
gi4504347	Hemoglobin subunit alpha	15.2	15.49	2.672828838
gi169217452	HLA class II histocompatibility antigen, DRB1-4 beta chain	25.7	8.97	2.548467326
gi56682959	Ferritin heavy chain	21.2	5.46	2.376697349
gi66346695	Fibrillin-2	314.6	0.24	2.288948591
gi40786436	Eukaryotic translation initiation factor 4A1	46.1	2.46	2.094114969
gi87196339	Collagen, type VI, alpha 1	108.5	0.88	1.986732711
gi4557733	Latent transforming growth factor beta binding protein 2	194.9	4.17	1.959638153
gi169210992	Hypothetical protein LOC100131693	25.1	3.69	1.909014562
gi24797076	MHC, class II, DR beta 1	29.1	3.49	1.879322964
gi207028935	Microfibrillar-associated protein 2 isoform b	20.7	5.49	1.846061192
gi4502107	Annexin A5	35.9	6.25	1.785327852
gi53946395	Tenascin	240.7	2.04	1.758381294
gi4758606	Integrin-linked protein kinase	51.4	2.43	1.673110304
gi13775595	Tryptase alpha/beta 1	30.5	6.55	1.638626466
gi5453678	Epididymal secretory protein E1	16.6	10.60	1.515388592
gi4557759	Myeloperoxidase	83.8	1.34	0.557022924
gi28827801	Anterior gradient protein 3	19.2	6.63	0.475747013
gi54607120	Lactotransferrin	78.1	1.27	0.334947079
gi124248516	Alpha-defensin 1	10.2	19.15	0.272535866
gi53945878	Mucin-5B	596.3	1.11	0.246384026

### Further identification and quantitative analysis of MUC5B in tumor tissue

**Figure 3:**
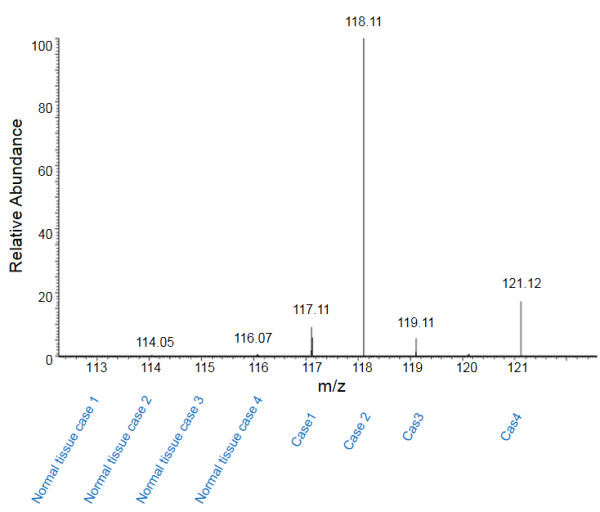
MS spectrum of mucin5B in pT1 lung adenocarcinomas and tumor-matched normal lung tissues.

**Figure 4:**
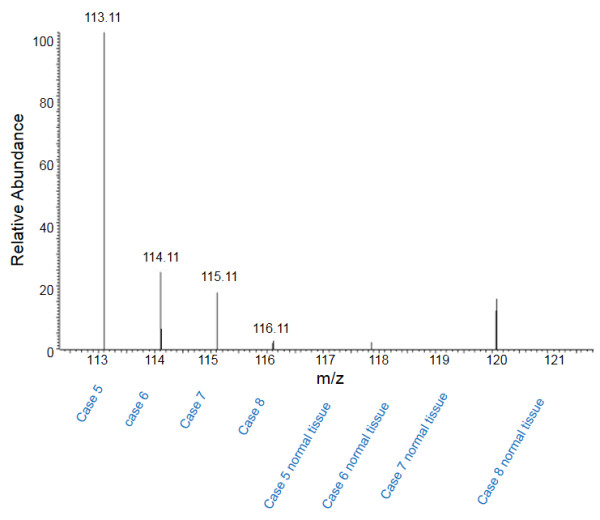
MS spectrum of mucin5B in pT2 lung adenocarcinomas and tumor-matched normal lung tissues.

We further analyzed the level of MUC5B in different stages of tumors and tumor-matched normal lung tissues using LC-MS/MS spectrum counts (Figure 3 and Figure 4). Our data demonstrated that the abundance of MUC5B in tumor tissue was significantly more than that in tumor-matched normal tissue regardless of tumor stages.

**Figure 5:**
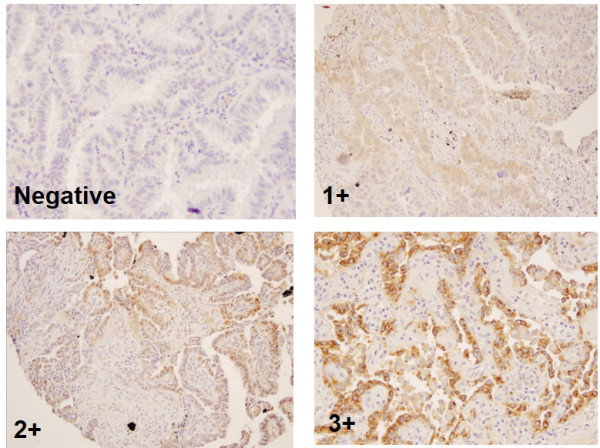
Immunostain of mucin 5B in lung adenocarcinomas.

To evaluate expression and cellular localization of MUC5B protein in lung adenocarcinoma tissues, we performed IHC using mouse anti-MUC5B monoclonal antibody (clone19.4E), which reacts with MUC5B and does not react with MUC5A or MUC5C. The overall expression of MUC5B in 45 lung adenocarcinoma tumors is summarized in Figure 5 and Table 3. Of 45 tumors, 13 cases (28.9%) were negative for MUC5B expression, 8 cases (17.8%) were weakly positive, 13 cases (28.9%) and 11 cases (24.4%) were focally and intensely positive. The predominant location of MUC5B in tumor cells was in the cytoplasmic membrane. Among different subtypes of adenocarcinomas, MUC5B was detected in 25% (1 of 4 cases) of adenocarcinomas with lepidic patterns, 62.5% (10 of 16 cases) of adenocarcinoms with papillary features, 66.7% (4 of 6 cases) of true papillary adenocarcinomas, 84.6% (11 of 13 cases) of adenocarcinomas NOS, and 100% (6 of 6 cases) of mucinous adenocarcinomas. In normal lung parenchyma, alveolar macrophages showed some cytoplasmic staining of MUC5B.

**Table 3:** Immunochemical stain of mucin 5B in lung adenocarcinomas

**Pathologic stage**	**IHC**	**IHC**	**IHC**	**IHC**
	**Staining**	**Staining**	**Staining**	**Staining**
	**Negative**	**1+**	**2+**	**3+**
pT1 (n = 24)	5 (20.8%)	4 (16.7%)	7 (29.2%)	8 (33.3%)
pT2 (n = 18)	6 (33.3%)	3 (16.7%)	6 (33.3%)	3 (16.7%)
pT3/4 (n = 3)	2 (66.7%)	1 (33.3%)		
Total (n = 45)	13 (28.9%)	8 (17.8%)	13 (28.9%)	11(24.4%)

## Discussion

The lung parenchyma and alveolar space are complex structures that contain numerous cellular proteins. In addition, lung cancer involves numerous genetic abnormalities with turn-on and turn-off expression at different stage of the tumor. In our study, in order to increase the detection of tumor associated proteins, different pathological stages of tumor tissues were included. Using the iTRAQ labeling approach, a total of 1288 peptides from 210 proteins were identified and quatified. Among them, 22 proteins showed greater than 1.5-fold differences between stage pT1 and pT2 tumors. A subset of proteins, such as fibrillin-2, ferritin, eukaryotic translation initiation factor 4A1 (eIF-4A1), annexin A5, mucin-5B (MUC5B), alpha-defensin 1 and anterior gradient protein 3 (hAG 3), are interesting.

In our study, the detection of MUC5B in tumor tissue is particularly interesting. Mucins (MUC) are a family of high molecular weight, heavily glycosylated proteins. In human, more than 20 mucin genes and related proteins are identified [19-21]. They can be divided into two major groups, membrane-associated mucin proteins including MUC1, MUC3, MUC4, MUC12, MUC13, MUC16 and MUC17; and secreted mucin proteins including both gel-forming such as MUC2, MUC5AC, MUC5B, MUC6 proteins, and non-gel-forming MUC7 protein [19-21]. Both types of mucins play important roles in the regulation of cellular functions such as formation of physical barrier, maintenance homeostasis of extracellular environment, and regulation of cell growth and differentiation. In cancer cells, mucin proteins also play important roles in tumor cell growth, invasion and metastasis [20]. Overexpression of MUC1 has been associated with potential risk of metastasis of colon cancer [30], pancreatic cancer [31] and oral squamous cell carcinoma [32]. The expression of MUC2 has been identified in pancreatic and liver cancer [33]. MUC4 protein contains an EGF (epidermal growth factor)-like motif on the extracellular domain and has been linked to the EGF signaling pathway [34,35]. It has been reported that main mucin genes in lung trachoebronchial tree are *MUC5B*, *MUC5AC*, and *MUC4*[21]. Among them, MUC5B is the major gel-forming mucin which forms a protective matrix in the airway. In normal adult lung parenchyma, the expression of MUC5B follows a distinct pattern. It is predominately expressed in the mucus producing cells in the large airway; the intensity of its expression is decreased from tracheobronchus towards the bronchioles, with no expression in small bronchioles and pneumocytes [36]. A few recent studies have shown that MUC5B is also frequently expressed in lung mucinous adenocarcinoma, and the expression of MUC5B is inversely correlated with the differentiation of the tumor [37]. Abnormal expressions of MUC1, MUC5 and MUC6 have also been related to the aggressive behavior of lung adenocarcinoma [22,23,38]. In our study, we found elevated expression of MUC5B in lung adenocarcinoma by iTRAQ labeling and further validated by IHC using lung adenocarcinoma TMAs. In the iTRAQ labeling study, it seems that there is a loss of MUC5B expression in higher stage of tumors (stage II lung adenocarcinomas), however, we will not be able to draw a conclusion due to a small set of cases in our study. We further performed an IHC assay using mouse anti-MUC5B monoclonal antibody (clone19.4E) to evaluate the expression of MUC5B using TMA. The monoclonal antibody in our study reacts with MUC5B, but does not react with MUC5A or MUC5C. We found that the expression of MUC5B was detected in 32 of 45 tumors (71.1%). Of 32 tumors, 8 cases (17.8%) were weakly positive, 13 cases (28.9%) and 11 cases (24.4%) were focally and intensely positive. The predominant location of MUC5B in tumor cells was in the cytoplasmic membrane. Among different subtypes of adenocarcinomas, MUC5B was detected in 25% (1 of 4 cases) of adenocarcinomas with lepidic patterns, 62.5% (10 of 16 cases) of adenocarcinoms with papillary features, 66.7% (4 of 6 cases) of true papillary adenocarcinomas, 84.6% (11 of 13 cases) of adenocarcinomas NOS, and 100% (6 of 6 cases) of mucinous adenocarcinomas. Our data demonstrates that MUC5B is abnormally expressed by most of tumors, and suggests that it may be a potential biomarker for lung adenocarcinomas.

Furthermore, we also found several proteins were differentially expressed in tumors, such as fibrillin-2, ferritin, hypothetical protein LOC100131693, eIF4A, annexin A5, anterior gradient-2 (hAG-2) and alpha-defensin-1. Fibrillin-2 is the key component of human elastic fiber in extracellular matrix. Aberrant methylation of its gene has been found in 53% of lung adenocarcinoms and has been correlated with large tumor size, nodal metastasis and advanced tumor stage in NSCLC [39]. Ferritin is an iron-binding protein; elevated serum level has been detected in advanced lung cancer patients, overexpression of this protein has been found in tumor tissue and has been reported to correlate with nodal metastasis in NSCLC [40]. Hypothetical protein LOC100131693 belongs to a group of proteins that are not described at the protein level but rather predicted from cDNA sequences [41]. It is of special interest as it may represent a new marker or marker for development of tumor vaccine, particularly when found in tumor tissue. eIF4A plays an important role in the regulation of eIF4E-eIF4G complex during the eukaryotic translation initiation in the AKT-mTOR signal pathway, which involves cell growth, angiogenesis, invasion, survival [42]. Annexin A5 is a phospholipid binding protein and binds to phophatidylserine. During early events of apoptosis, annexin A5 binds to phosphatidylserine on the cell membrane, thus regulates programmed cell death. It has been found to be overexpressed in p-XSC treated mice lung adenocarcinoma [43]. Interestingly, the use of isotopic labeled annexin A5 as a clinical tool for visualization of cell death has been suggested to be important in monitoring patient’s response to chemotherapy [44]. The human anterior gradient-2 (hAG-2) gene has been proposed an oncogene for NSCLC. The hAG-2 is expressed in lung squamous cell carcinoma [45]. In the screening of breast tumor tissue using reverse transcription-PCR and immunohistochemistry with affinity purified anti-hAG-2 antibodies, Liu et al. reported that the presence of hAG-2 mRNA and protein were associated with estrogen alpha (ERA)-positive carcinomas [46]. There were no differences in the mean latency periods of tumor formation when an expression vector bearing hAG-2 cDNA was introduced into benign rat mammary tumor cells, but metastases occurred at high rates in the lung of animals receiving the hAG-2 transfectants (77–92% of animals with primary tumors, compared with 0% in the control groups). Their results suggest that hAG-2 may be a potential marker related to cancer metastasis. Alpha-defensin-1, also known as the human neutrophil peptides (HNP1-3), is a small cationic peptide found in neutrophil granules. Alpha-defensin showed cytotoxicity to various types of eukaryotic and tumor cells. The mechanism of alpha-defensin induced cell damage and death involves release of cytochrome c from mitochondria, which is the key event of mitochondria-mediated apoptosis; it has been found to inhibit the growth of human lung adenocarcinoma xenograft in nude mice [47]. The Biological role of these proteins in lung adenocarcinomas need to be further investigated.

A recent study by Kikuchi T, et al [48] has shown that 3513 proteins were identified from pooled lung adenocarcinoma tissues using the shotgun proteomic method, and they further validate a few candidate proteins using multiple reactions monitoring MS, IHC staining and Western blotting analysis. In comparison to their study, we have also identified similar proteins in tumor tissues using iTRAQ labeling approach, such as eIF, collagen type VI, tenascin, anterior gradient protein, MUC5B, and hemoglobin subunit alpha BAL. In our recent study of lung airway fluid, we have further identified a subset of proteins derived from tumor tissue in the airway fluid specimens [29]. Taken together, these findings are important and suggest that unique tumor-derived proteins can be identified in both lung parenchyma and airway fluid, and some of these proteins could be further tested and validated as potential biomarkers for the detection of lung adenocarcinomas.

In summary, our data demonstrate that a large number of protein changes during the lung cancer disease process can be identified by quantitative proteomic approaches. However, the potential role of these identified proteins need to be further validated and characterized. The study of protein expression in lung cancers is also important for understanding the disease process, assessing the tumor progression, and customizing therapeutic modalities for individual patients.

## Consent

The use of human tumor tissues and related pathological information was approved by the Johns Hopkins Institutional Review Board.

## Competing interests

The authors declare that they have no competing interests.

## Authors’ contributions

YL carried out the MS study and analyzed data, and wrote the manuscript, XW and MH Ao performed IHC studies and analyzed data, EG, FA and HZ provided critical supports for the study and critics for the manuscript. QKL wrote and was responsible for the manuscript. All authors read and approved the final manuscript.
